# Technology-based teaching to support health students’ clinical skills in stroke recovery: a scoping review

**DOI:** 10.1186/s12909-026-08709-7

**Published:** 2026-02-17

**Authors:** Kylie Bower, Katharine Scrivener, Sarah Larkins, Catherine Seaton, Karen Carlisle

**Affiliations:** 1 Murtupuni Centre for Rural and Remote Health, James Cook University, Mount Isa, Australia; 2 Alfred Health and Monash University, Melbourne, Australia; 3 James Cook University, Townsville, Australia

**Keywords:** Stroke recovery, Technology-based teaching and learning, Health students, Facilitators, Student-resourced services

## Abstract

**Background:**

Rural stroke survivors face barriers to accessing rehabilitation, and the challenge is likely to grow as acute medical advances improve survival rates. Student-resourced services are used to support urban stroke rehabilitation, and there is growing interest in the use of technology-based teaching approaches to enable health student skill development. There is potential for technology-based student teaching techniques to support rural stroke rehabilitation capacity, but this is currently unexplored. This scoping review aimed to understand the characteristics of technology-based teaching techniques that support health students to develop clinical skills for stroke recovery.

**Methods:**

The PRISMA-ScR approach guided a comprehensive search of five databases and grey literature. Studies were included if the participants were health students who engaged in technology-based teaching activities of less than 12 hours’ duration, addressing clinical skills relevant to stroke recovery. Studies from all geographical, clinical, and educational contexts were considered, irrespective of study design. Nine data elements were summarised descriptively, and content analysis was applied to describe facilitators of technology-based teaching, using an inductive approach.

**Results:**

Forty-eight studies were included in the review. Studies were predominantly published in the last 5 years (54%), were of lower methodological quality (69%), and featured medical (43%), physiotherapy (27%) or nursing students (27%) in educational (71%) - not clinical - settings. Features of technology-based approaches were identified as facilitators of teaching and learning: online or face-to-face delivery, engaging multimedia design, clinical skill practice and interaction or feedback. Only one paper demonstrated the use of technology-based teaching approaches in a rural, student-resourced stroke service.

**Conclusion:**

The identified facilitatory features can inform the design of a technology-based learning approach for student-resourced stroke rehabilitation, ensuring alignment with teaching and learning theories, defined student learning outcomes and the needs of stroke survivors. Implications for rural services are unclear. Research is warranted to explore the gap in technology-based learning for rural student-resourced stroke rehabilitation services.

**Supplementary Information:**

The online version contains supplementary material available at 10.1186/s12909-026-08709-7.

## Background

Rural residents’ stroke risk and incidence are higher than their urban counterparts’ in many countries [1–4]. At least 80% of rural stroke survivors may have ongoing physical, psychosocial, informational, and support needs that require rehabilitation [5, 6]; however, their experience of local stroke recovery services may be fragmented and lacking in expertise [7, 8]. This contributes to geographical inequity in stroke care [3], particularly where stroke incidence is low or variable [9, 10]. The availability of rural stroke rehabilitation services is a challenge, requiring innovative solutions and enhanced resourcing [9]. A service innovation that warrants consideration is a student-resourced model.

Student-resourced rehabilitation is an emerging approach in several countries, predominantly for underserved communities and people with neurological conditions, including stroke [11]. In rural and remote settings, student-resourced models have improved access to under-resourced health services [12], and specifically for Indigenous communities [13]. In urban settings, student-resourced stroke rehabilitation services have demonstrated benefits, including positive learning experiences for allied health students, perceived benefits for stroke survivors with unmet needs [14, 15], and improvements in physical performance outcomes [16]. Collectively, these studies [11–15] highlight the potential mutual value for allied health students and stroke survivors who participate in student-resourced stroke rehabilitation services in rural settings.

### Teaching and learning approaches in student-resourced services

Teaching and learning factors are critical to the effectiveness of student-resourced stroke rehabilitation. This includes high-quality orientation, clinical supervision, and opportunities for peer-assisted learning [15]. However, innovative solutions may be needed to enable these conditions in a rural context where generalist skills are required. For example, online education activities have been used to connect students on rural clinical placement to external clinical educators for skill development [12]. Wearable technology has remotely connected educators to nursing students with audio and visual instruction to practice clinical procedural tasks [17]. These examples point to the value of exploring a combination of face-to-face and technology-enhanced teaching and learning opportunities – known as ‘blended learning’ - to support student-resourced rural stroke recovery services.

### Blended learning in student-resourced services

Garrison and Kanuka [18] define blended learning as the effective and intentional integration of online and face-to-face learning opportunities. Blended learning in clinical education placements has involved technologies such as videoconferencing, blogging, chat rooms, social media and web-based resources [19]. For developing students’ stroke clinical skills, technologies have included multimedia e-learning tools [20], interactive computer-assisted instruction, instructor-led videotapes [21], and freely available online videos [22]. Benefits include improved psychological and behavioural learning outcomes; for example, academic performance, clinical skills, cooperation with peers, self-regulation towards learning, and satisfaction and engagement with learning [23]. However, challenges are also well-documented, predominantly the availability of information technology skills and infrastructure, particularly in under-resourced and rural locations [19, 23, 24].

This scoping review aims to identify the characteristics of technology-based learning activities that support health students’ clinical skills for stroke recovery, which could inform a blended-learning approach for rural clinical placements. The review focuses on facilitators of learning as design considerations, acknowledging that barriers to blended learning have been documented elsewhere [19, 23, 24]. The term ‘rural’ is used to refer to regional, rural and remote health services, because definitions of terms vary across international stroke literature [4]. The review objectives align with the technology-based learning model [25], including:

1. To describe characteristics in terms of context (rural/urban/educational/clinical), disciplines, learning objectives, teaching tools and learning strategies.
2. To describe the facilitators of teaching and learning that could inform a rural blended learning strategy.
3. To summarise the quality of existing research.

## Methods

The scoping review methodology was guided by the Preferred Reporting Items for Systematic reviews and Meta-Analyses extension for Scoping Reviews (PRISMA-ScR) checklist [26], and Joanna Briggs Institute guides for scoping reviews [27, 28]. A review protocol was developed with input from the research team, but this was not registered.

### Inclusion criteria

#### Participants

Participants were limited to Australian University of Department of Rural Health disciplines [29]. This was to optimise the transferability of results to the student clinical placement context in rural Australia.

#### Concept

Included sources described the implementation of technology-based teaching and learning elements (either a tool, activity or application) that could be delivered in less than 12 h, either synchronously or asynchronously, within a stroke-related clinical placement. Both stroke and brain injury applications were included because of their shared clinical skill set.

Sources were excluded where only the development, not the implementation, of a teaching and learning activity was described. Activities of more than 12 hours’ duration were excluded because time can be a barrier to using technology-based learning activities in a clinical placement [19].

#### Context

Teaching and learning activities from clinical and educational learning environments were included to identify activities that could be transferred to a clinical placement context. A pilot search revealed no sources that were specific to the Australian rural context; therefore, sources from world-wide metropolitan and non-metropolitan locations were included.

#### Type of sources

The review included primary studies and reviews with qualitative, quantitative, and mixed-method designs. Sources from library databases and grey literature were sought to provide a broad description of this emerging field [27].

### Search strategy

A search strategy was developed in Medline (OVID) in consultation with a university librarian. The key search terms that aligned with Person-Concept-Context elements (students, health disciplines, blended or online learning, and stroke or traumatic brain injury) were used to identify initial sources that met inclusion criteria. From the titles, abstracts, and index terms of these sources, keywords and Medical Subject Headings (MeSH) were identified. The search was not limited by study design, language, or date of publication. The Medline search was adjusted for CINAHL, Emcare, ERIC and Scopus (see Additional file 1: Search strategy table). Initial searches were completed in July 2024.

Grey literature searches were conducted using simplified key terms (student, education, stroke) because more refined search terms, similar to those used in databases, returned very few results. Grey literature sources included MedNar database, academic repositories (Health System Evidence, MedlinePlus), government sites (.edu.au, .ac.nz, and .gov), organisational websites (Stroke Ed, Informme, Stroke Engine, Chest Heart & Stroke Scotland, World Stroke Academy, Angels Initiative) and Google. Initial searches were completed in November 2024. Both database and grey literature searches were updated in March 2025.

### Evidence screening and selection

Search results were imported to Endnote 20, duplicates removed, then titles and abstracts were screened independently by two authors (KB & CS). Disagreements were discussed, and inclusion criteria were refined iteratively. The same authors independently reviewed full-texts using the refined criteria, and reasons for exclusion were recorded. Discrepancies between authors were resolved by consensus.

### Critical appraisal of evidence sources

Although critical appraisal of sources is not generally required for scoping reviews [27] this step was completed to understand the quality of the research in this emerging field. The first author appraised all studies using the Mixed Methods Appraisal Tool [30]. To support rigor, a third of the papers (*n* = 16) were appraised in consultation with an author with expertise in mixed methods, and an author with expertise in quantitative methods, and discrepancies were resolved with advice from a senior researcher. All criteria were met by 15 papers (31%) (see Additional file 2: Summary of critical appraisal).

### Data extraction – charting and data items

A charting table was piloted and refined with input from the research team [28] to extract: publication year and country, disciplines involved, learning objectives addressed, blended or online approach, synchronous or asynchronous activities, technology-based element, clinical or educational environment, rural or metropolitan setting, and facilitatory teaching strategies. A second reviewer (CS) completed data extraction for 10 (21%) of the full texts. Discrepancies in results were resolved by consensus, leading to the refinement of some definitions with the wider team, and presented in the data extraction guidance sheet (see Additional file 3: Data extraction guidance sheet).

### Data analysis and presentation

Facilitatory teaching strategies were synthesised using basic qualitative content analysis with an inductive approach [28]. Verbatim statements were extracted into an Excel spreadsheet, keywords were highlighted, and initial descriptors were allocated. A coding framework was developed to organise these data and reviewed by the team. The remaining data were summarised descriptively in Excel using percentages and frequency counts [28]. All extraction was completed by one author (KB), with regular review by at least two other team members. The purpose was to summarise the data, rather than to interpret the evidence [28]. Qualitative and quantitative data are presented in Table 1.

**Table 1:** Data extraction table. Table summarising the nine quantitative data elements and the one qualitative data element that were extracted from the included papers

	Author	Year	Country	Discipline	Learning objective	Blended / online / F2F	Synchronous / Asynchronous	Technology:	Clinical: Y/N	Rural: Y/N	Facilitators
1	Ada et al	2003	AUS	PT	Ax	B	S	Videos	N	N	MM/CE/F2F
2	Alverson et al	2004	USA,AUS	Med	Rx	O	S	Virtual reality simulation, chat function	N	N	O/I/CE
3	Baccin et al	2020	BRA	Nurs	Ax, Rx	B	S&A	Smartphone app, online resources	Y	N	O/CE
4	Bai & Lavin	2016	USA	Nurs	Ax, Rx	B	S	Online resources, discussion, virtual simulation	N	N	O/I/CE
5	Bernhardt et al	2001	AUS	PT	Ax	B	S	Videos	N	N	F2F/MM/I
6	Bleske	2022	USA	Pharm	Ax	B	S&A	Online modules, smartphone app	Y	Y	O/CE
7	Bondoc & Wall	2015	USA	PT/OT	Rx	B	S&A	Videos	N	N	I/CE
8	Bornkamm et al	2021	DEU	Med	Ax	B	S&A	Videos	N	N	O/F2F/MM/I/CE
9	Britnell et al	2014	NZL	Nurs	Ax, Rx	B	S	Tablet device, online clinical tool	Y	N	O/CE
10	Chan & Cheng	2017	HKG	SP	Ax	B	S	Videos	N	N	MM/I
11	Cooper et al	2007	GBR	PT/OT/ES	Kn	O	A	Online module	N	N	O/M/I
12	Curry et al	2024	CAN	Med	Kn	O	A	Online module	Y	N	O/M
13	Dancer et al	2017	USA	Nurs	Ax	O	A	Online module vs DVD	N	N	MM
14	Deutsch et al	2023	USA	PT	Ax	B	Both	Online clinical tool	N	N	O/M
15	Ding et al	2023	SGP	Med	Ax	O vs B	A	Online module & discussion, virtual practice	N	N	O/MM/I/CE
16	Feuntes et al	2023	CHL	Med	Kn	O vs B	Both	Video-conference, online virtual simulator	N	N	I/CE
17	Finch et al	2020	AUS	SP	PWA	B	Both	Video-conference telepractice of clinical skill	Y	N	O/CE
18	Frey et al	2021	USA	Med	Ax	B	Both	Online module, interactive videos	N	N	O/F2F/MM/CE
19	Gawlik et al	2015	USA	Nurs/Med/Pharm/PT	Ax, Rx	B	Both	Online module, online clinical tool	Y	N	O/CE
20	Groth et al	2018	DEU	Med	Ax	O vs F2F	Both	Online module	N	N	O/F2F/MM
21	Hartsgrove	2023	USA	Med	Att	B	S	Online lectures & virtual encounters with patients, videos	Y	N	O/MM/CE
22	Johnson et al	2020	USA	Med/Nurs	IPP	B	Both	Online modules, videos and slides	N	N	O/F2F/MM/CE
23	Kamel et al	2021	CAN	Med	Ax	O	Both	Online lecture, Q&A & videos	N	N	O
24	Kang & Kang	2022	KOR	Med/nurs	IPP, CR	O	S	Mixed reality simulation (holographic), videoconference	N	N	O/I/CE
25	Kim & Shin	2024	KOR	Nurs	Ax	B	Both	Extended reality with a head-mounted display device	N	N	O/MM/CE
26	Lavin & Bai	2015	USA	Nurs	Kn, Att	B	S	Videos, online discussion, online simulation	N	N	O/I/CE
27	Lee & Son	2023	KOR	Nurs	Ax	B vs F2F	Both	Virtual reality simulation	N		O/CE
28	Liu et al	1997	CAN	OT/PT	Rx	B vs F2F	S	Videos	N	N	F2F/CE
29	Loebel et al	2024	USA	Med	Kn, Att	O	S	Online lectures with interactive features, simulation and videos	Unclear	N	O/MM
30	MacKenzie	1990	USA	Nurs	Kn	B	A	Videos	Y	N	MM
31	Maeno et al	2004	JPN	PT	Rx	B	S	Videos, videoconference, live feedback on skill performance	N	N	O/I/CE
32	Malhotra et al	2024	GBR	Med	Ax	O	S	Online lecture, videoconference	Y	N	O
33	Mills	2020	AUS	OT/SP/Diet	IPP	B	A&S	Video-based simulation	N	N	F2F/MM/I/CE
34	Newcomer et al	2022	USA	OT	Kn	O	A	Online modules with discussion & videos	N	Y	O/MM
35	Pourmand et al	2018	USA	Med	Ax	O	A	Online module (videos, lecture)	Y	N	O/M
36	Power et al	2020	AUS	OT	PWA	O vs F2F	A&S	Online lecture, videos, reflective questioning, passive skill building	N	N	O/MM
37	Power et al	2024	AUS	OT/PT/SP/Other	PWA	O	A&S	Online modules & videoconference with videos & discussion	N	N	O/MM/I
38	Preston et al	2012	AUS	PT	Rx	B	A&S	Online videos & instruction sheets)	N	N	O/MM
39	Preston et al	2020	AUS	PT	Rx	O	A&S	Online resource (videos, downloadable instruction sheets)	Y	N	O
40	Rajan & Pandit	2022	GBR	Med	Kn	O	A	Online module (interactive, clinical cases), webpage	Y	N	O/MM/I/CE
41	Rochette et al	2023	CAN	OT/PT/SP/Phar/Nurs/Med	Kn	O	A	Online website	Y	N	O
42	Sarfo et al	2021	GHA	Med	Ax	O	S	Video-conference, smart phone or tablet with headsets, online skill practice with feedback	Y	N	O
43	Scholten	2021	AUS	SP	Kn	B	S	Online module (interactive multimedia, video, narration)	N	N	MM
44	Stephens et al	2013	GBR	Rad/Nurs	IPP	B	A&S	Online learning system, online website creation	N	N	O/I
45	Suppan et al	2021	CHE	Med	Ax	O	A	Online module (videos, interactive multimedia & questions with feedback)	N	N	O/MM
46	Vakilian et al	2022	IRN	Med	Kn	O	A&S	Online video, interactive multimedia	N	N	MM
47	Veneri & Gannotti	2011	USA	PT	CR	B	A&S	Online module with videos	N	N	O/MM
48	Worm & Jensen	2013	DNK	Med	Rx	O	A	Online module (lecture, videos, peer chat) & online text book	N	N	O/MM/I

*AUS* Australia, *BRA* Brazil, *DEU* Germany, *NZL* New Zealand, *HKG* – Hong Kong, *GBR* United Kingdom of Great Britain and Northern Ireland, *CAN* Canada, *SGP* Singapore, *CHL* Chile, *KOR* Republic of Korea, *JPN* Japan, *GHA* Ghana, *CHE* Switzerland, *IRN* Iran, *DNK* Denmark, *PT* Physio or physical therapy, *Med * Medical, *Nurs* Nursing, *Pharm* Pharmacy, *OT* Occupational therapy, *SP* Speech pathology, *ES* Exercise science, *Diet* Dietetics, *Rad* Radiology, *Ax* Assessment, *Rx* treatment, *Kn* Knowledge, *IPP* Interprofessional practice, *PWA* Communicate with people with aphasia, *Att* Attitudes, *CR* Clinical reasoning or critical thinking, *B* Blended, *O* Online, *F2F* Face to face, *MM* Multimedia design, *I* Feedback or interaction, *CE* Clinical experience or simulation

## Results

### Selection of sources

**Fig. 1:**
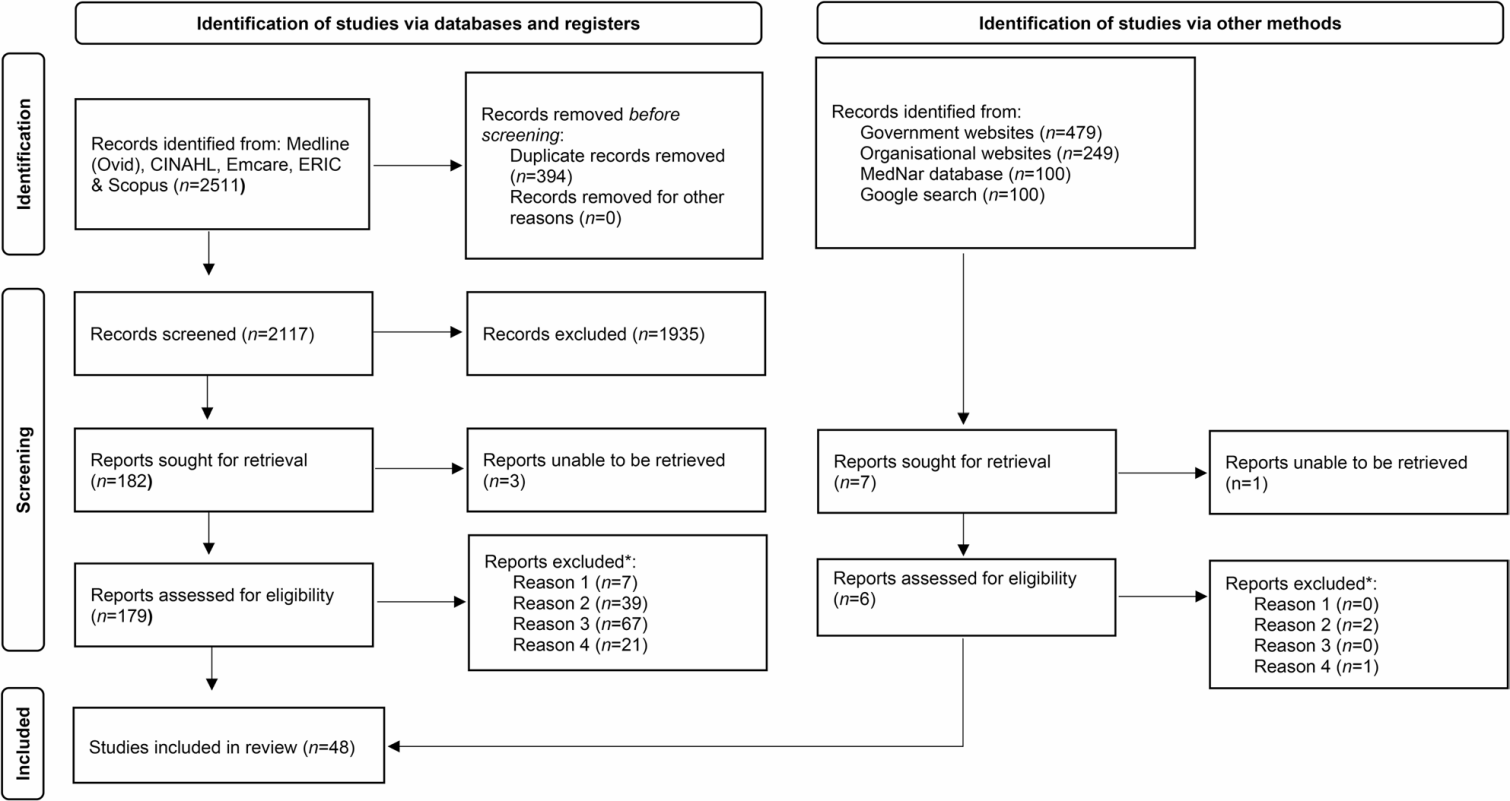
PRISMA flow chart. PRISMA flow chart summarising the process of identification, screening and inclusion of sources

Searches of five databases identified 2511 papers. Endnote was used to remove 394 duplicates, leaving 2117 papers. These titles and abstracts were screened, as well as 928 sources that were identified in grey literature. After screening, 182 full texts were sought from the database papers and seven from grey literature sources, of which three and one were unable to be retrieved, respectively. Full texts of 185 papers were screened, excluding 137 for four reasons as detailed in the PRISMA chart (see Fig. 1). Forty-eight papers remained for inclusion in the scoping review, and data extraction for these papers are summarised in Additional file 4.

*Reasons for exclusion: Reason 1: participant is not a UDRH health discipline; Reason 2: is not an evaluation of a blended or on-line teaching / learning strategy; Reason 3: application of the teaching / learning strategy to stroke or brain injury is not articulated; Reason 4: teaching / learning strategy is not clearly feasible in a clinical placement (e.g. >12 h duration).

### Quantitative reporting

- Characteristics of sources of evidence.

Publication dates spanned 34 years, and 54% (*n* = 26) were published in the last five years (2020–2024). Studies were based in 16 countries, predominantly USA (*n* = 16) and Australia (*n* = 10).

b. Characteristics of teaching and learning activities.

Sixteen disciplines of health students were involved across the 48 studies. Medical students were most frequently studied (*n* = 21), followed by physiotherapy or physical therapy students (*n* = 13), nursing students (*n* = 13) and interdisciplinary groups (*n* = 10).

The student learning objectives addressed assessment skills (*n* = 21), treatment planning or skills (*n* = 12), knowledge of the condition (*n* = 11), interprofessional practice (*n* = 4), communicating with people with aphasia (*n* = 3), clinical reasoning (*n* = 2), and attitudes (*n* = 3).

A blended approach was reported in 28 studies, online in 22, and two studies compared online to blended approaches. Synchronous activities were described in 37 papers (77%), asynchronous in 34 studies (71%) and almost half (*n* = 23, 48%) used both.

The most frequently reported technology was multimedia learning, featuring in 39 studies. This included videos, electronic learning modules (online or on CD-ROM), online learning systems, and multimedia slide presentations. Less frequently reported technologies included online discussion (*n* = 11), videoconferencing (*n* = 8) and patient simulations or encounters in video-based, 3D, virtual, extended or mixed reality format (*n* = 11). Few studies employed online clinical tools or apps (*n* = 5), online clinical skill practice (excluding simulations) (*n* = 4), mobile devices (*n* = 4) or the creation of a website as the learning activity.

An educational setting (*n* = 34) was more prevalent than a clinical environment (*n* = 14). Thirty-seven studies were conducted in metropolitan settings, although two of these discussed how learnings could be applied in a rural setting. Only one paper described a rural or remote clinical education setting [31].

### Content analysis of facilitating features

All studies identified facilitating features of the learning approach (online versus blended) or strategies (including multi-media, clinical experience or practice, and interaction with peers or educators).

- Learning approach.

Online delivery was a frequently reported facilitatory feature, within an approach that was either blended or exclusively online. Online delivery enhanced geographical access [32–42], enabled flexible and student-led learning [32, 33, 36, 40, 43–46], and increased efficiency of teaching resources or processes [41, 47–50]. When accessed through a mobile device, an online learning tool was used to scaffold patient-facing clinical performance [31, 49, 51, 52]. Some studies concluded that online environments were equally or more efficacious than face-to-face learning environments for learning outcomes [50, 53].

In contrast, other studies highlighted the benefits of face-to-face learning opportunities or limitations with online approaches. Face-to-face learning enabled direct clinical educator feedback [43, 54], clinical simulation, or skill practice [43, 55–58]. An online clinical learning tool, ‘Physiotherapy eSkills Training Online’, demonstrated less value in a clinical placement versus a classroom environment, possibly because clinical placement educators did not intentionally integrate the resource into the learning approach [46, 59].

b. Learning strategies.

Multimedia design was frequently stated as a facilitator of learning. Videos were used widely to enhance visual learning by, for example, observing authentic clinical scenarios [45, 47, 60], enabling ‘passive skill building’ or modelling [50]. Interactive features enhanced active learning using, for example, drag-and-drop, multi-choice, click-and-point activities [37], polls [32], and questions for reflection and clinical reasoning [61]. One study demonstrated that e-modules with more sophisticated visual and interactive features led to stronger learning outcomes when compared to modules with less sophisticated features [62].

Clinical experience or practice – either online or face-to-face - was a facilitator. Technology enabled this through virtual simulation programs [51, 63–65], online guided mental rehearsal of a psychomotor skill [66], and online encounters with patients [42, 67]. Other studies emphasized face-to-face experiences to support student-patient rapport [55] and enhanced student experience [57]. Supplementary skills-based training and feedback was recommended to strengthen an online communication partner training package [36].

Interaction with peers or educators was highlighted. Performance feedback supported skill accuracy [43, 54]. This was equally effective when provided by an educator as by automated means in a virtual simulation of clinical decision-making [64]. Feedback or social interaction enhanced student satisfaction with learning experiences [37, 40, 62] although this did not necessarily translate into stronger learning outcomes [37]. The social experience of a teaching activity was particularly valued in an interprofessional context to build interprofessional knowledge and skills [40, 58, 65, 68].

## Discussion

This scoping review identified 48 texts describing technology-based teaching and learning activities for health students to obtain stroke clinical knowledge and skills. Publication trends indicated rapid growth in the last 5 years, primarily in the USA and Australia; however, methodological quality was generally low. Facilitators of learning varied according to context, including online versus face-to-face delivery, multimedia design, clinical skill practice, and interaction with educators or peers. The key characteristic of teaching and learning activities was heterogeneity: multiple disciplines were targeted for various learning objectives, using a range of technology-based tools and teaching strategies. Few papers featured a clinical setting, and a rural location was described in one lower-quality study [31]. Therefore, implications for rural student clinical placements are unclear. This discussion will explore these findings in the context of broader literature, highlighting potential considerations for rural stroke clinical contexts.

The prevalence of recent publications reflects a growing interest in technology-based learning for students in stroke recovery, consistent with literature across health education [23, 25, 69], and specifically for medical, nursing, physiotherapy, and interdisciplinary students [19, 70, 71]. Results may need to be extrapolated for application to less-represented allied health disciplines such as occupational therapy and social work, which are pivotal to holistic stroke recovery services, but frequently under-represented in rural stroke services [10].

The facilitators of learning identified in this review align with learning theories and other reviews of blended and technology-based learning, regarding face-to-face versus online delivery, multimedia design, clinical skill practice and interactivity [21, 25, 70, 72–74]. Face-to-face learning enables student discussion, psychomotor practice, or problem-based learning [25, 70], consistent with theories of Situated Learning, Experiential Learning, Active Learning and Social Cognitive Theory [75]. Online learning is considered a norm in health professional education [71, 74] and rural health practice [76] and creates successful learning experiences for health students [21, 72, 73] when it is designed with strong theoretical foundations [77] and principles of multimedia learning [78]. The benefits of interaction and feedback can be maximised in online learning using social learning software and social media [79], break-out rooms, text-chat, and questions [76]. This, together with face-to-face clinical educator supervision and support [80], role modelling, peer learning [81], and interprofessional encounters [82] supports a sense of belonging for students, which in turn can influence students’ intention to work rurally [83]. These findings are consistent with Social Cognitive and Community of Practice theories, whereby groups of students with a common purpose develop shared knowledge, values, and skills [75]. Teaching and learning theory, concepts, and models must drive effective resource development for health students [84], specifically in rural settings [76], and where students are leading clinical service delivery [85].

A challenge for stroke clinical educators will be to determine which learning strategies to choose from the broad range identified in this review. The heterogeneity is driven partly by ongoing technology advances [19, 71], by the context-specific nature of blended learning that considers the learners’ needs and educators’ capacity [18], and by individual stroke survivors’ therapy needs [7]. Consequently, clinical educators will need to curate blended learning approaches for multiple stakeholders, warranting a co-creation approach [86] as exemplified elsewhere for students working in delirium [87].

Other challenges may include students’ skills in, and acceptance of, technology-based learning [19, 59]. This could be mitigated by clarifying the purpose and relevance of learning tasks and enabling students to practice using technology-based tools in advance [19].

A further challenge is that rural stroke services are typically under-resourced [3, 9] in stroke clinical expertise [9], information technology skills and infrastructure [19, 23]. This significantly threatens the capacity of rural services to design and implement co-created, evidence-based and theoretically sound blended learning resources, consistent with barriers to digital transformation in under-resourced communities worldwide [24]. Potential innovative solutions were demonstrated in this review to supplement local clinical expertise, for example, using online guided mental rehearsal for psychomotor skill practice [66], and using online clinical tools to guide clinical assessments [47], stroke risk screening, and patient education [49]. Other external asynchronous learning resources include online videos [22] and websites such as informme.org.au, strokeed.com, strokeengine.ca. Videoconference or social media could facilitate synchronous learning activities with external stroke specialists [19]. Solutions such as these warrant further high-quality research in the rural clinical placement setting.

### Strengths and limitations

Strengths of this review include the range of databases and grey literature sources searched, the number of studies identified, the critical appraisal, and the synthesis of teaching approaches across disciplines and locations. A limitation is the scarcity of identified studies based in rural and clinical contexts, meaning that the aim of the review was only partially met. The analysis did not extract data about the experiences of stroke survivors who were involved in the learning activities, the teaching and learning theories or models that underpinned approaches, nor the outcomes. The omission of barriers in data extraction may have biased the review towards positive features of technology-based learning. The discussion has highlighted barriers identified in existing literature to balance this. Each of these missing elements could be a focus for future scoping reviews. More recent technological tools were not addressed, such as artificial intelligence, which will become integral to health professional education [69]. Consistent with other reviews in the field, most sources included in the review were of low methodological quality [19, 70, 88], and originated from a limited number of more developed countries [11, 19, 70, 88]. Collectively, these factors limit the generalisability of findings, particularly to diverse rural clinical settings.

## Conclusions

This scoping review identified a proliferation of studies of technology-based learning for health students in stroke recovery; however, studies lacked methodological rigour and were not specific to rural contexts. Among the heterogeneity of teaching strategies, several prominent design considerations were identified: selecting online versus face-to-face learning tasks, optimising multimedia design, ensuring there is clinical skill practice and enabling high-quality interaction with educators and peers. These elements should be chosen intentionally to align with the stakeholders involved, including students, educators and stroke survivors, and should be based on theories and models of teaching and learning. In a rural context, innovative solutions may need to account for resource challenges, such as technology infrastructure and skill, and stroke clinical expertise. Research is warranted to explore the gap in high-quality technology-based learning for student-resourced stroke rehabilitation services, particularly in a rural context.

## Supplementary Information

Supplementary Material 1. Search strategy table. Description of data: Table listing keywords and MESH used for five data base searches

Supplementary Material 2. Data extraction guidance sheet. Description of data: table detailing the definitions of data that were extracted for quantitative and qualitative analysis

Supplementary Material 3. Summary of critical appraisal using the Mixed Methods Appraisal Tool. Description of data: table listing the results of the critical appraisal of included studies

## Data Availability

The dataset supporting the conclusions of this article is included within the additional files (see Additional file 4).
