# Supplementary material for: Technology-based teaching to support health students’ clinical skills in stroke recovery: a scoping review
Source: BMC Med Educ. 2026 Feb 17;26:472. doi: 10.1186/s12909-026-08709-7 (PMC13014826; doi:10.1186/s12909-026-08709-7)
Supplement: Supplementary file 1 — Supplementary Material 1. Search strategy table. Description of data: Table listing keywords and MESH used for five data base searches [file 12909_2026_8709_MOESM1_ESM.docx]

Additional file 1: Search strategy table

|  | | **Population: Students** | **Population:**  **Health discipline** | **Concept:**  **Online or blended learning** | **Context:**  **Stroke or Traumatic brain injury (TBI)** |
| --- | --- | --- | --- | --- | --- |
| **Medline** | **Keywords** | Student* OR undergraduate* OR under-graduate* OR pre-registration OR preregistration OR post-graduate* OR postgraduate OR “Graduate education” | Health OR “Allied Health” OR “Occupational Therap*” OR “Physical Therap*” OR physiotherap* OR “pharmac*” OR “Speech patholog*” OR “Speech therap*” OR “Speech language patholog*” OR “Speech language therap*” OR “Exercise physiolog*” OR “exercise therap” OR dietic* OR dietit* OR dietet* OR nutritionist* OR medic* OR Nurs* OR podiatr* OR psycholog* OR “Social work*” | “Digital health” OR “e-learn*” OR “technology-enhanced learn*” OR “computer-based train*” OR “computer-assisted instruction” OR “distance educat*” OR “videoconferenc*” OR “self-directed learn*” OR “stroke course*” OR “remote learn*” OR “blended learn*” OR “flipped classroom” OR “online learn*” OR internet OR online OR ‘world wide web’ OR ‘world-wide-web’ | Stroke OR “neurologic* rehabilitation” OR “cognitive training” OR “stroke rehabilitation” OR apoplexy OR “brain vascular accident*” OR CVA* OR “cerebral stroke*” OR “cerebrovascular accident*” OR "cerebral vascular accident*" OR “basal ganglia cerebrovascular disease” OR “neurorehabilitation” OR “brain infarct*” OR “brain stem infarct*” OR “brain Ischemia” OR “cerebral infarct*” OR “cerebrovascular trauma” OR "intracranial embolism and thrombosis" or “intracranial hemorrhages” OR “h*emorrhagic stroke” OR “isch*emic stroke” OR “thrombotic stroke” OR “lateral medullary syndrome” OR “brain injur*” OR “*phasia” OR “*praxia” OR “hemianop*” OR “neurologic* exam*” OR “stroke patient*” OR “Head Injuries” OR “neurological impairments” OR aphasia OR “brain hemisphere functions” OR “communication disorders” OR “language impairments” OR “perceptual impairments” OR “speech impairments” OR “speech improvement” OR “speech evaluation” OR “cognitive ability” OR “short term memory” |
|  | **MESH** | exp Students/ | exp allied health personnel/ or faculty, medical/ or faculty, nursing/ or health educators/ or exp medical staff/ or exp nurses/ or exp nursing staff/ or nutritionists/ or occupational therapists/ or personnel, hospital/ or exp medical staff, hospital/ or nursing staff, hospital/ or pharmacists/ or physical therapists/ or exp physicians/ or psychotherapists/ or allied health occupations/ or occupational therapy/ or physical therapy specialty/ or speech-language pathology/ or Podiatry/ or Nutritionists/ or Health personnel/ or Social Workers/ or exp Social Work/ or nutritional sciences/ or dietetics/ or public health/ or exp preventive medicine or exp Nursing/ | problem-based learning/ or education, distance/ or exp education, professional/ or gamification/ or exp self-directed learning as topic/ or exp simulation training/ | brain injury, chronic/ or exp brain injuries/ or exp basal ganglia cerebrovascular disease/ or exp brain ischemia/ or exp cerebrovascular trauma/ or exp "intracranial embolism and thrombosis"/ or exp intracranial hemorrhages/ or exp stroke/ or exp Neurological Rehabilitation/ |

|  | | **Population: Students** | **Population:**  **Health discipline** | **Concept:**  **Online or blended learning** | **Context:**  **Stroke or TBI** |
| --- | --- | --- | --- | --- | --- |
| **CINAHL** | **Keyword** | As per medline |  |  |  |
|  | **MESH** | (MH "Students+") | (MH "Dietitians") OR (MH "Exercise Physiologists") OR (MH "Habilitation Personnel+") OR (MH "Occupational Therapists+") OR (MH "Physical Therapists+") OR (MH "Speech-Language Pathologists") OR (MH "Nutritionists") OR (MH "Rehabilitation Personnel+") OR (MH "Social Workers") OR (MH "Faculty, Allied Health") OR (MH "Faculty, Medical") OR (MH "Faculty, Nursing") OR (MH "Medical Staff+") OR (MH "Novice Clinicians+") OR (MH "Nurses+") OR (MH "Personnel, Health Facility+") OR (MH "Pharmacists") OR (MH "Physicians+") OR (MH "Podiatrists") OR (MH "Rural Health Personnel") OR (MH "Allied Health Professions+") OR (MH Education, Health Sciences+) OR (MH "Public Health Nutrition") OR (MH "Rehabilitation+") OR (MH "Specialties, Allied Health") OR (MH "Pharmacy and Pharmacology+") OR (MH "Podiatry") OR (MH "Social Work+") OR (MH "Medicine+") OR (MH "Stroke Nurses") OR (MH "Nurses") OR (MH "Psychology") OR (MH "Occupational Therapy+") OR (MH "Speech-Language Pathology") OR (MH "Rehabilitation Science") OR (MH "Exercise Physiology+") OR (MH "Public Health Nutrition") OR (MH "Nutritionists") OR (MH "Dietetics") OR (MH "Psychologists") | (MH "Educational Technology") OR (MH "Experiential Learning") OR (MH "Audiorecording") OR (MH "Motion Pictures") OR (MH "Slides") OR (MH "Television") OR (MH "Videorecording") OR (MH "Fieldwork") OR (MH "Gamification") OR (MH "Computer-Assisted Instruction") OR (MH "Computer Simulation+") OR (MH "Patient Simulation") OR (MH "Vignettes") OR (MH "Teaching Methods, Clinical+") OR (MH "Problem-Based Learning") OR (MH "Self-Directed Learning") OR (MH "Education, Non-Traditional+") | (MH "Brain Injuries+") OR  (MH "Stroke+") OR (MH "Intracranial Hemorrhage+") OR (MH "Stroke Patients") |

|  | | **Population: Students** | **Population:**  **Health discipline** | **Concept:**  **Online or blended learning** | **Context:**  **Stroke or TBI** |
| --- | --- | --- | --- | --- | --- |
| **Emcare** | **Keyword** | As per medline |  |  |  |
|  | **MESH** | exp Students/ | exp allied health personnel/ or faculty, medical/ or faculty, nursing/ or health educators/ or exp medical staff/ or exp nurses/ or exp nursing staff/ or nutritionists/ or occupational therapists/ or personnel, hospital/ or exp medical staff, hospital/ or nursing staff, hospital/ or pharmacists/ or physical therapists/ or exp physicians/ or psychotherapists/ or allied health occupations/ or occupational therapy/ or physical therapy specialty/ or speech-language pathology/ or Podiatry/ or Nutritionists/ or Health personnel/ or Social Workers/ or exp Social Work/ or nutritional sciences/ or dietetics/ or public health/ or exp preventive medicine or exp Nursing/ | problem-based learning/ or education, distance/ or exp education, professional/ or gamification/ or exp self-directed learning as topic/ or exp simulation training/ | brain injury, chronic/ or exp brain injuries/ or exp basal ganglia cerebrovascular disease/ or exp brain ischemia/ or exp cerebrovascular trauma/ or exp "intracranial embolism and thrombosis"/ or exp intracranial hemorrhages/ or exp stroke/ or exp Neurological Rehabilitation/ |

|  | | **Population: Students** | **Population:**  **Health discipline** | **Concept:**  **Online or blended learning** | **Context:**  **Stroke or TBI** |
| --- | --- | --- | --- | --- | --- |
| **ERIC** | **Keywords** | “Higher education” OR “clinical experience” OR “college students” OR “adult students” | Rehabilitation OR “Health Occupations” OR “Professional Personnel” OR Therapy OR “Physical Therapy” OR “Occupational therapy” OR “allied health occupations” OR “allied health occupations education” OR “Health personnel” OR “Health services” OR “Medical education” OR “Medical services” OR “Medical schools” OR “Graduate medical education” OR “Health Sciences” OR Medicine OR Physicians OR “Clerkships” OR “Professional Occupations” OR Kinesiology OR “Physical Therapy” OR “Speech Therapy” OR Pharmacy OR dietetics OR podiatry OR “Speech language pathology” OR “Exercise physiology” OR neuropsychology OR “nursing education” OR “Nursing students” OR nurses OR psychologists OR psychology OR “psychological services” OR “Social work” | “blended learning” OR “computer assisted instruction” OR “computer uses in education” OR “distance education” OR “educational strategies” OR “educational technology” OR “electronic learning” OR “experimental teaching” OR gamification OR “instructional innovation” or “integrated activities” OR “online courses” OR “open educational resources” OR “teaching machines” OR “technology integration” OR telecourses OR “training methods” or videoconferencing OR “virtual universities” OR “virtual classrooms” or “web based instruction” OR “flipped classroom” OR “audiovisual aids” OR “instructional films” OR “interactive video” OR “Learner controlled instruction” OR “learning management systems” OR “multimedia instruction” OR hybrid | MAINSUBJECT.EXACT.EXPLODE("Technology Uses in Education") OR MAINSUBJECT.EXACT.EXPLODE("Blended Learning") |
|  | **MESH** | MAINSUBJECT.EXACT.EXPLODE("Higher Education") OR MAINSUBJECT.EXACT.EXPLODE("Clinical Experience") OR MAINSUBJECT.EXACT.EXPLODE("College Students") OR MAINSUBJECT.EXACT.EXPLODE("Adult Students") | MAINSUBJECT.EXACT.EXPLODE("Allied Health Occupations") OR MAINSUBJECT.EXACT.EXPLODE("Health Personnel") OR MAINSUBJECT.EXACT.EXPLODE("Health Occupations") OR MAINSUBJECT.EXACT.EXPLODE("Therapy") OR MAINSUBJECT.EXACT.EXPLODE("Medicine") OR MAINSUBJECT.EXACT.EXPLODE("Speech Language Pathology") OR | MAINSUBJECT.EXACT.EXPLODE("Technology Uses in Education") OR [MAINSUBJECT.EXACT.EXPLODE("Blended Learning")](https://www.proquest.com/recentsearches.recentsearchtabview.recentsearchesgridview.scrolledrecentsearchlist.checkdbssearchlink:rerunsearch/B570958E2B6B4C97PQ/None/$N?site=eric&t:ac=RecentSearches) | MAINSUBJECT.EXACT.EXPLODE("Head Injuries") OR MAINSUBJECT.EXACT.EXPLODE("Aphasia") |

|  | | **Population: Students** | **Population:**  **Health discipline** | **Concept:**  **Online or blended learning** | **Context:**  **Stroke or TBI** |
| --- | --- | --- | --- | --- | --- |
| **Scopus** | **Keywords** | “Higher education” OR “clinical experience” OR “college students” OR “adult students” OR Student* OR undergraduate* OR under-graduate* OR pre-registration OR preregistration OR post-graduate* OR postgraduate OR “Graduate education” | "Rehabilitation team" OR "Health Occupations" OR "Professional Personnel" OR "Physical Therapy" OR "allied health occupations" OR "Health personnel" OR "Health services" OR "Medical education" OR "Medical services" OR "Medical schools" OR "Graduate medical education" OR "Health Sciences" OR Medicine OR Physicians OR Clerkships OR "Professional Occupations" OR Kinesiology OR "Speech Therapy" OR Pharmacy OR dietetics OR "Exercise physiology" OR neuropsychology OR "nursing education" OR "Nursing students" OR psychology OR "psychological services" OR "Allied Health" OR "Occupational Therap*" OR "Physical Therap*" OR physiotherap* OR "pharmac*" OR "Speech patholog*" OR "Speech therap*" OR "Speech language patholog*" OR "Speech language therap*" OR "Exercise physiolog*" OR "exercise therap" OR dietic* OR dietit* OR dietet* OR nutritionist* OR medic* OR Nurs* OR podiatr* OR psycholog* OR "Social work*" | “Digital health” OR “e-learn*” OR “technology-enhanced learn*” OR “computer-based train*” OR “computer-assisted instruction” OR “distance educat*” OR “videoconferenc*” OR “self-directed learn*” OR “stroke course*” OR “remote learn*” OR “blended learn*” OR “flipped classroom” OR “online learn*” OR internet OR online OR "world wide web" OR "world-wide-web" OR “computer uses in education” OR “distance education” OR “educational strategies” OR “educational technology” OR “electronic learning” OR “experimental teaching” OR gamification OR “instructional innovation” or “integrated activities” OR “online courses” OR “open educational resources” OR “teaching machines” OR “technology integration” OR telecourses OR “training methods” or videoconferencing OR “virtual universities” OR “virtual classrooms” or “web based instruction” OR “audiovisual aids” OR “instructional films” OR “interactive video” OR “Learner controlled instruction” OR “learning management systems” OR “multimedia instruction” OR hybrid | Stroke OR “neurologic* rehabilitation” OR “cognitive training” OR “stroke rehabilitation” OR apoplexy OR “brain vascular accident*” OR CVA* OR “cerebral stroke*” OR “cerebrovascular accident*” OR "cerebral vascular accident*" OR “basal ganglia cerebrovascular disease” OR “neurorehabilitation” OR “brain infarct*” OR “brain stem infarct*” OR “brain Ischemia” OR “cerebral infarct*” OR “cerebrovascular trauma” OR "intracranial embolism and thrombosis" or “intracranial hemorrhages” OR “h*emorrhagic stroke” OR “isch*emic stroke” OR “thrombotic stroke” OR “lateral medullary syndrome” OR “brain injur*” OR “*phasia” OR “*praxia” OR “hemianop*” OR “neurologic* exam*” OR “stroke patient*” OR “Head Injuries” OR “neurological impairments” OR aphasia OR “brain hemisphere functions” OR “communication disorders” OR “language impairments” OR “perceptual impairments” OR “speech impairments” OR “speech improvement” OR “speech evaluation” OR “cognitive ability” OR “short term memory” |
