# Supplementary material for: Technology-based teaching to support health students’ clinical skills in stroke recovery: a scoping review
Source: BMC Med Educ. 2026 Feb 17;26:472. doi: 10.1186/s12909-026-08709-7 (PMC13014826; doi:10.1186/s12909-026-08709-7)
Supplement: Supplementary file 2 — Supplementary Material 2. Data extraction guidance sheet. Description of data: table detailing the definitions of data that were extracted for quantitative and qualitative analysis [file 12909_2026_8709_MOESM2_ESM.docx]

Additional file 3: Data extraction guidance sheet

|  | Author | Eg Smith; Smith & Hunt; Smith et al. |
| --- | --- | --- |
| Quantitative data points | 1. Year | The year the article was published. |
|  | 1. Location | The country / countries where the study was conducted. |
|  | 1. Type of evidence source & methodology | Primary research (qualitative / RCT / non-randomised CT / quantitative descriptive / mixed methods).  Evidence syntheses (narrative / systematic / scoping / rapid)  Other (discussion articles, editorials, theses). |
|  | 1. Participants | Discipline of health student (medical / nursing / pharmacy / allied health (specify)). |
|  | 1. Stroke-related learning objective | Knowledge of condition (eg presentation of stroke, neuroanatomy of stroke, assessment and intervention options)  Assessment (eg neurological examination, CT scan interpretation, movement analysis)  Treatment planning or skills (eg  Clinical reasoning OR critical thinking  Communicating with PWA (people with aphasia)  Interprofessional practice understanding or performance  Other (eg attitudes) |
|  | 1. Teaching / learning strategy – blended or online environment | Blended learning ('the thoughtful integration of classroom face-to-face learning experiences with online learning experiences’ (18)). Online learning (no learning activities occur face-to-face). |
|  | 1. Teaching / learning strategy – synchronous or asynchronous | Synchronous – learning occurs ‘in real time’.  Asynchronous – strategy occurs in a self-directed manner, at a time of the students’ choosing. |
|  | 1. Technology-based tools | For example, instant response systems, mobile phones or tablet, online learning systems (eg Google Classroom, Moodle), social media platform (eg FaceBook or YouTube), learning modules (online or CD-ROM), multimedia learning (eg videos), simulations in virtual reality / mixed reality / 3D, online discussions / chat, clinical tool or app, online game, online clinical practice, other. |
|  | 1. Clinical setting specified | Y = patients are involved in the teaching or learning strategy for the clinical benefit of the patient (ie instead of seeing a health professional). This may occur in a clinical setting such as a hospital or a university therapy clinic.  N = patients are involved primarily for the educational benefit of the students (eg clinical demonstration, clinical practice or OSCE). |
|  | 1. Rural or remote application specified | Y = author clearly articulates how this learning event could be applied in a rural or remote context. |
| Qualitative data point | 1. Facilitators of teaching / learning | What feature of the teaching / learning strategy is identified in the discussion as a facilitator of effective student learning & / or clinical performance? Eg blended or online approach / technology-based element / synchronous or asynchronous strategy / duration / clinical vs educational setting. |
