# Supplementary material for: Technology-based teaching to support health students’ clinical skills in stroke recovery: a scoping review
Source: BMC Med Educ. 2026 Feb 17;26:472. doi: 10.1186/s12909-026-08709-7 (PMC13014826; doi:10.1186/s12909-026-08709-7)
Supplement: Supplementary file 3 — Supplementary Material 3. Summary of critical appraisal using the Mixed Methods Appraisal Tool. Description of data: table listing the results of the critical appraisal of included studies [file 12909_2026_8709_MOESM3_ESM.docx]

Additional file 2: Summary of critical appraisal using the Mixed Methods Appraisal Tool (29)

| Citation | Study design | Criteria met  (see described below) |
| --- | --- | --- |
| Power et al 2020 | RCT | 5/5^a^ |
| Rajan et al 2022 | RCT | 5/5^a^ |
| Ding et al 2023 | RCT | 4/5^a^ |
| Vakilian et al 2022 | RCT | 4/5^a^ |
| Worm et al 2013 | RCT | 4/5^a^ |
| Fuentes et al 2023 | RCT | 3/5^a^ |
| Suppan et al 2021 | RCT | 3/5^a^ |
| MacKenzie et al 1990 | RCT | 3/5^a^ |
| Curry et al 2024 | RCT | 1/5^a^ |
| Dancer et al 2017 | RCT | 1/5^a^ |
| Liu et al 1997 | RCT | 1/5^a^ |
| Bornkamm et al 2021 | Non-RCT | 5/5^b^ |
| Lee & Son 2023 | Non-RCT | 5/5^b^ |
| Pourmand et al 2018 | Non-RCT | 5/5 |
| Preston et al 2012 | Non-RCT | 5/5^b^ |
| Bernhardt et al 2001 | Non-RCT | 4/5^b^ |
| Malhotra et al 2024 | Non-RCT | 4/5^b^ |
| Power et al 2024 | Non-RCT | 4/5^b^ |
| Ada et al 2003 | Non-RCT | 3/5^b^ |
| Baccin et al 2020 | Non-RCT | 3/5^b^ |
| Hartsgrove et al 2023 | Non-RCT | 3/5^b^ |
| Newcomer et al 2022 | Non-RCT | 3/5^b^ |
| Scholten et al 2021 | Non-RCT | 3/5^b^ |
| Chan & Chen 2017 | Non-RCT | 2/5^b^ |
| Sarfo et al 2021 | Non-RCT | 1/5^b^ |
| Groth et al 2018 | Non-RCT | 0/5^b^ |
| Bai & Lavin 2016 | Non-RCT | 0/5^b^ |
| Alverson et al 2004 | Non-RCT | 0/5^b^ |
| Loebel et al 2024 | Quantitative descriptive | 3/5^c^ |
| Britnell et al 2014 | Quantitative descriptive | 3/5^c^ |
| Johnson et al 2020 | Quantitative descriptive | 2/5^c^ |
| Kamel et al 2021 | Quantitative descriptive | 2/5^c^ |
| Kang & Kang 2022 | Quantitative descriptive | 2/5^c^ |
| Lavin & Bai 2015 | Quantitative descriptive | 1/5^c^ |
| Gawlik et al 2015 | Quantitative descriptive | 0/5^c^ |
| Maeno et al 2004 | Quantitative descriptive | 0/5^c^ |
| Bondoc & Wall 2015 | Mixed methods | 5/5^d^ |
| Deutsch et al 2023 | Mixed methods | 5/5^d^ |
| Finch et al 2020 | Mixed methods | 5/5^d^ |
| Kim & Shin 2024 | Mixed methods | 5/5^d^ |
| Mills et al 2020 | Mixed methods | 5/5^d^ |
| Preston et al 2020 | Mixed methods | 5/5^d^ |
| Rochette et al 2023 | Mixed methods | 5/5^d^ |
| Veneri et al 2011 | Mixed methods | 5/5^d^ |
| Cooper et al 2007 | Mixed methods | 4/5^d^ |
| Frey et al 2021 | Mixed methods | 3/5^d^ |
| Stephens et al 2013 | Mixed methods | 2/5^d^ |
| Bleske et al 2022 | Mixed methods | 1/5^d^ |
| Mixed Methods Appraisal Tool criteria (29):   1. RCT criteria: 1. Is randomisation appropriately performed? 2. Are the groups comparable at baseline? 3. Are there complete outcome data? 4. Are outcome assessors blinded to the intervention provided? 5. Did the participants adhere to the assigned intervention? 2. Non-RCT criteria: 1. Are the participants representative of the target population? 2. Are measurements appropriate regarding both the outcome and the intervention (or exposure) 3. Are there complete outcome data? 4. Are the confounders accounted for in the design and analysis? 5. During the study period, is the intervention administered (or exposure occurred) as intended? 3. Quantitative descriptive criteria: 1. Is the sampling strategy relevant to address the research question? 2. Is the sample representative of the target population? 3. Are the measurements appropriate? 4. Is the risk of nonresponse bias low? 5. Is the statistical analysis appropriate to answer the research question? 4. Mixed methods criteria: 1. Is there an adequate rationale for using a mixed methods design to address the research question? 2. Are the different components of the study effectively integrated to answer the research question? 3. Are the outputs of the integration of qualitative and quantitative components adequately interpreted? 4. Are divergences and inconsistencies between quantitative and qualitative results adequately addressed? 5. Do the different components of the study adhere to the quality criteria of each tradition of the methods involved? | | |
